# Highly Prevalent Multidrug-Resistant *Salmonella* From Chicken and Pork Meat at Retail Markets in Guangdong, China

**DOI:** 10.3389/fmicb.2018.02104

**Published:** 2018-09-10

**Authors:** Lina Zhang, Ying Fu, Zhiying Xiong, Yeben Ma, Yihuan Wei, Xiaoyun Qu, Hongxia Zhang, Jianmin Zhang, Ming Liao

**Affiliations:** ^1^National and Regional Joint Engineering Laboratory for Medicament of Zoonoses Prevention and Control, Guangzhou, China; ^2^Key Laboratory of Zoonoses, Key Laboratory of Animal Vaccine Development, Ministry of Agriculture, Guangzhou, China; ^3^Key Laboratory of Zoonoses Prevention and Control of Guangdong Province, Guangzhou, China; ^4^College of Veterinary Medicine, South China Agricultural University, Guangzhou, China

**Keywords:** *Salmonella*, retail meat, prevalence, antibiotic resistance, ESBLs, PFGE

## Abstract

This study aimed to investigate the prevalence, serotype distribution, and antibiotic resistance, and to characterize the extended spectrum β-lactamases (ESBLs) producing *Salmonella* isolates from chicken and pork meats from retail markets in Guangdong province, China. A total of 903 retail meat samples (475 chicken and 428 pork meats) were obtained from six cities (Guangzhou, Shenzhen, Heyuan, Shaoguan, Foshan, and Yunfu) of Guangdong province between May 2016 and April 2017. High levels of *Salmonella* contamination were detected in chicken (302/475, 63.6%) and pork (313/428, 73.1%). Thirty-eight serotypes were identified in 615 detected *Salmonella*, and the serotypes varied greatly between chicken and pork samples. Agona (55/302, 18.2%), Corvallis (45/302, 14.9%), Kentucky (38/302, 12.6%), Mbandaka (32/302, 10.6%) was the dominant serotypes in chicken samples. However, Typhimurium (78/313, 24.9%), Rissen (67/313, 24.1%), Derby (66/313, 21.1%), and London (48, 15.3%) were the most common in pork samples. High rates of antibiotic resistance were found to sulfisoxazole (468/615, 76.1%), tetracycline (463/615, 75.3%), ampicillin (295/615, 48.0%), and ofloxacin (275/615, 44.7%). Notably, antimicrobial susceptibility tests identified resistance to polymyxin B (12/615, 2.0%) and imipenem (3/615, 0.5%). Multidrug-resistance (MDR) was detected in *Salmonella* isolated from chicken (245/302, 81.1%) and pork (229/313, 73.2%). The resistance rate of different *Salmonella* serotypes varied widely. Especially, isolates such as Typhimurium, Agona, *C*orvallis and Kentucky exhibited highly resistance to antibiotics. The MDR rate of *Salmonella* isolates from chicken was significantly higher than that from pork isolates (*P* < 0.05). Twenty-one *Salmonella* isolates were identified as ESBLs-producing, covering six *Salmonella* serotypes and displaying different pulse field gel electrophoresis (PFGE) genotypes. *Bla_OXA-*1*_* was the dominant ESBLs gene (9/21, 42.9%), followed by *bla_CTX-M-*55*_* (5/21, 23.8%). This study indicated that *Salmonella* was widespread in chicken and pork from retail markets in Guangdong province and the isolates showed high multidrug-resistance, especially the known multidrug-resistant *Salmonella* serotypes. Therefore, it is important to focus on *Salmonella* serotypes and strengthen the long-term monitoring of MDR *Salmonella* serotypes in animal-derived foods.

## Introduction

*Salmonella* is a foodborne pathogen that causes morbidity and mortality worldwide (Scallan et al., 2011). Until now, more than 2,600 serovars have been identified among *Salmonella* worldwide and almost all *Salmonella* can cause illness in humans and animals (Guibourdenche et al., 2010). In China, about 70–80% of foodborne disease outbreaks are caused by *Salmonella*, and the majority of diseases are associated with the ingestion of contaminated livestock and poultry products (Wang et al., 2007). Animal-derived foods, especially chicken and pork, are considered the major reservoirs of *Salmonella* dissemination (Vo et al., 2006; Jackson et al., 2013).

At the same time, antibiotic resistance of *Salmonella* is also one of the most important public health problems worldwide. In recent years, studies have shown that because of long-term antibiotic use during animal breeding, antibiotic resistance has markedly increased. Multidrug-resistant (MDR) *Salmonella* could pose a serious threat to humans through the food chain (Barza, 2002; Lai et al., 2014). In particular, extended-spectrum β-lactamases (ESBLs)-producing *Salmonella* have been frequently isolated from food animals in many countries, including China (Dahms et al., 2015; Ben Said et al., 2016; Ibrahim et al., 2016; Zhao et al., 2017).

For animal-derived foods, tracking the source of *Salmonella* infections can help to identify potential problems in food production, processing, preparation, or the sales process to prevent the introduction of other pathogens into food (Swaminathan et al., 2006; Russell et al., 2014). Guangdong is a livestock production and consumption province; furthermore, Guangdong is China’s foremost livestock production area. In 2009, laboratory-based surveillance for *Salmonella* infection was established in Guangdong Province, which mainly monitors the prevalence and outbreak of *Salmonella* in various hospitals (Deng et al., 2012). However, limited information on surveillance studies of *Salmonella* in animal-derived foods from retail markets in Guangdong is available, and there is a lack of epidemiological data.

The main aim of this study was to determine the prevalence, serotype distribution, antibiotic resistance of *Salmonella*, and the phenotype and genotype of ESBLs-producing *Salmonella* isolated from chicken and pork meat from retail markets in Guangdong. The results could lay the foundation for follow-up research for public health security and food safety problems caused by *Salmonella*.

## Materials and Methods

### Sample Collection

A total of 903 retail meat samples (including 475 chicken and 428 pork sample) were randomly collected from retail markets in six cities (Guangzhou, Shenzhen, Heyuan, Shaoguan, Foshan, and Yunfu; chosen from three retail markets in each district) of Guangdong province between May 2016 and April 2017. Sampling methods were as follows: In Guangzhou City, different regions were sampled monthly; in other cities, samples were taken monthly from three selected retail markets. Samples were taken of different chicken types (i.e., whole chickens and chopped chickens), pork types (i.e., dressed pork) and during different seasons (i.e., spring, summer, autumn and winter). Each sample was placed in a sterile plastic sample bag and transported to the laboratory on ice. All samples were analyzed on the day of their collection.

### *Salmonella* Isolation and Identification

*Salmonella* isolation and identification was performed according to the Standard ISO-6579 (International Organization for Standardization, 2002) method, as described previously (Yang et al., 2010; Ren et al., 2016) with some modifications. Briefly, 25 g of samples was placed into a sterile plastic bag containing 225 mL of buffered peptone water and shaken for 3 min. The suspension was then cultivated at 37°C in a Shaker at 100 rpm for 6–8 h and then 1 mL of the suspension was added to 10 mL Tetrathionate Broth and Rappaport–Vassiliadis soya broth and incubated at 42°C for 18–24 h, separately. After selective enrichment, the suspensions were streaked onto xylose lysine tergitol 4 agar and CHROMagar^TM^ incubated at 37°C over night. Isolates with typical *Salmonella* phenotypes were further confirmed using API 20E test strips (bioMerieux, Marcy-l’Etoile, France).

### *Salmonella* Serotyping

All the *Salmonella* isolates were serotyped according to the White Kauffmann Le Minor scheme or National Food Safety Standard-Food microbiological examination: *Salmonella* (GB 4789.4-2016) by slide agglutination, using specific O and H antisera (S&A Reagents Lab Ltd., Bangkok, Thailand).

### Antimicrobial Susceptibility Testing and Screening for ESBLs Strains

All *Salmonella* isolates were tested for antibiotic susceptibility using the Kirby-Bauer disk diffusion method, and the results were interpreted according to the standards of the Clinical and Laboratory Standards Institute (CLSI, 2013).

Agar diffusion assays were performed on Muller-Hinton agar with disks containing 18 different antibiotic agents (Oxoid, Basingstoke, United Kingdom): ampicillin 10 μg (AMP), amoxicillin-clavulanic acid 30 μg (AMC), cefotaxime 30 μg (CTX), ceftazidime 30 μg (CAZ), cefepime 5 μg (FEP), gentamicin 10 μg (GEN), streptomycin 10 μg (STR), amikacin 30 μg (AMK), sulfamethoxazole-trimethoprim 23.75/1.25 μg (SXT), sulfonamides 300 μg (S3), nalidixic acid 30 μg (NAL), ofloxacin 5 μg (OFX), ciprofloxacin 5 μg (CIP), florfenicol 30 μg (FFC), chloramphenicol 30 μg (CHL), imipenem 10 μg (IPM), polymyxin B 300 IU (PB), and tetracycline 30 μg (TET). *Escherichia coli* ATCC 25922 and ATCC 35218 were used as quality control organisms. Isolates exhibiting resistance to three or more antibiotic classes were defined as MDR.

The *Salmonella* isolates were screened for ESBLs production using cefotaxime and cefotaxime (30 μg)/clavulanic acid (10 μg) disks and ceftazidime and ceftazidime (30 μg)/clavulanic acid (10 μg) disks (Oxoid) according to the double-disk synergy test method (Emery and Weymouth, 1997). Phenotypic presence of ESBLs of the isolates was determined by detecting enhancement of the diameter of the inhibition zone of the clavulanate disk and corresponding β-lactam antibiotic disk. If there was difference of ≥5 mm between the inhibition zone of the clavulanic acid and the β-lactam antibiotic disk, the isolate confirmed as having a positive ESBLs phenotype (CLSI, 2013).

### PCR Detection and DNA Sequencing

Salmonella isolates showing ESBLs phenotypes were further confirmed by PCR and DNA sequencing. ESBLs encoding genes detected included *bla_TEM_*, *bla_CTX-M_*, *bla_CMY_*, *bla_OXA_*, *bla_PSE_*, and *bla_SHV_*. The primers for the ESBLs encoding gene and the reaction conditions were as previously described (Li et al., 2013; Nguyen et al., 2016; Qiao et al., 2017). The PCR products were stained with ethidium bromide, visualized under UV light after gel electrophoresis using 1% agarose, purified, and sent to Sangon Biotech Co., Ltd. (Shanghai, China) for sequencing. The ESBLs encoding gene fragments were analyzed and aligned at GenBank using the online BLAST software^1^.

### Pulse Field Gel Electrophoresis (PFGE)

Extended spectrum β-lactamases-producing *Salmonella* isolates were subtyped by PFGE to determine their genetic relatedness, according to the Pulse-Net protocol recommended by the CDC (Ribot et al., 2006). PFGE was preformed after digestion of the genomic DNA with the restriction enzyme XbaI, *Salmonella enterica* subsp. *enterica* serovar Braenderup (CDC no. H9812) was used as standard control strain. PFGE results were analyzed using BioNumerics Software (Version 5.1; Applied-Maths, Sint-Martens-Latem, Belgium).

### Statistical Analysis

The isolation rates and antibiotic resistance rates among the different food types and isolates were analyzed using the Chi-squared test in SPSS Statistics 18.0 software (SPSS Inc., United States). A *P*-value of <0.05 was considered statistically significant.

## Results

### Prevalence and Serotypes of *Salmonella*

In this study, a total of 615 *Salmonella* isolates (615/903, 68.1%) were recovered from the meat samples. High levels of *Salmonella* contamination were detected: 302 (302/475, 63.6%) in chicken samples and 313 (313/428, 73.1%) in pork samples (**Table 1**). The isolation rate from pork was higher than that from chicken (*P* < 0.05).

**Table 1:** Prevalence and serotypes of *Salmonella* isolated from chicken and pork meat at retail markets in Guangzhou, China.

Source of samples	No. of positive samples /No. of total sample(%)	Serovar of isolates (number of samples)	Number of Serovars
Chicken	302/475 (63.6)	Agona (55), Corvallis (45), Kentucky (38), Mbandaka (32), Braenderup (21), Enteritidis (21), Rissen (14), Indiana (12), Derby (9), London (9), Meleagridis (7), Typhimurium (6), Stanley (5), Cerro (5), Albany (4), Hadar (4), Senftenberg (3), Litchfield (3), Weltevreden (1), Anatum (1), Muenster (1), Infantis (1), Haifa (1), Rumford (1), Saint paul (1), Thompson (1), Schwarzengrund (1).	27
Pork	313/428 (73.1)	Typhimurium (78), Rissen (67), Derby (66), London (48), Meleagridis (13), Stanley (9), Give (4), Weltevreden (3), Krefeld (3), Newport (3), Mbandaka (2), Senftenberg (2), Anatum (2), Gold coast (2), Agona (1), Corvallis (1), Muenster (1), Infantis (1), Alachua (1), Bovismorbificans (1), Brandenburg (1), Chailey (1), K é douguo (1), Kottbus (1), Reading (1).	25
Total	615/903 (68.1)		38

Thirty-eight *Salmonella* serotypes were identified among the 615 *Salmonella* isolates (**Table 1**). More serovars were found in chicken samples (27 serovars) than in pork samples (25 serovars). The serotypes varied greatly between the chicken and pork samples. In the chicken samples, the most common serovars were Agona (55/302, 18.2%), Corvallis (45/302, 14.9%), Kentucky (38/302,12.6%), and Mbandaka (32/302, 10.6%); and in the pork samples they were Typhimurium (78/313, 24.9%), Rissen (67/313, 24.1%), Derby (66/313, 21.1%), and London (48, 15.3%). Importantly, serovar Rumford was detected in meat samples for the first time in China.

### Antimicrobial Susceptibility Testing

The 615 isolated strains were tested for their susceptibility to 18 antibiotics (**Table 2**). The most resistance was to sulfisoxazole (468/615, 76.1%) and tetracycline (463/615, 75.3%), followed by ampicillin (295/615, 48.0%), ofloxacin (275/615, 44.7%), and trimethoprim/sulfamethoxazole (248/615, 40.3%). All isolates were susceptible to cefepime, ceftazidime, amikacin, and amoxicillin-clavulanic acid. Notably, the results showed very low rates of resistance to polymyxins and carbapenem antibiotics, such as polymyxin B (12/615, 2.0%) and imipenem (3/615, 0.5%).

**Table 2:** Antibiotic resistance of *Salmonella* isolates from retail meats in Guangdong, China.

Antibiotic	Number of resistant isolates (%) from:		
	Chicken (*n* = 302)	Pork (*n* = 313)	Total (*n* = 615)
β-lactams			
Ampicillin	96 (31.8)	199 (63.6)	295 (48.0)
Amoxicillin/clavulanic acid	4 (1.3)	3 (1.0)	7 (1.1)
Cefotaxime	30 (9.9)	10 (3.2)	40 (6.5)
Ceftazidime	10 (3.3)	4 (1.3)	14 (2.3)
Cefepime	8 (2.6)	9 (2.9)	17 (2.8)
Aminoglycosides			
Gentamicin	34 (11.3)	66 (21.1)	100 (16.3)
Streptomycin	84 (27.8)	127 (40.6)	211 (34.3)
Amikacin	6 (2.0)	2 (0.6)	8 (1.3)
Sulfonamides			
Trimethoprim/sulfamethoxazole	103 (34.1)	145 (46.3)	248 (40.3)
Sulfisoxazole	212 (70.2)	256 (81.8)	468 (76.1)
Quinolones			
Nalidixic acid	112 (37.1)	65 (20.8)	177 (28.8)
Ofloxacin	192 (63.6)	83 (26.5)	275 (44.7)
Ciprofloxacin	37 (12.3)	37 (11.8)	74 (12.0)
Phenicols			
Florfenicol	142 (47.0)	96 (30.7)	238 (38.7)
Chloramphenicol	146 (48.3)	99 (31.6)	245 (39.8)
Carbapenems			
Imipenem	0	3 (1.0)	3 (0.5)
Polymyxins			
Polymyxin B	7 (2.3)	5 (1.6)	12 (2.0)
Tetracyclines			
Tetracycline	216 (71.5)	247 (78.9)	463 (75.3)

Resistance to ampicillin, tetracycline, streptomycin, sulfisoxazole, Florfenicol, Chloramphenicol, and Ofloxacin was frequently observed in the *Salmonella* serovars isolated (**Table 3**): 588 (95.6%) isolates were resistant to at least one antibiotic and 474 (77.1%) were MDR (**Table 4**). Resistance to 4–9 antibiotics was detected in 331 isolates (53.8%), whereas 57 isolates (9.3%) were resistant to ≥10 antibiotics. More of the serovars isolated from chicken showed MDR ≥3 (*n* = 245) than did the serovars isolated from pork (*n* = 229) (*P* < 0.05). The MDR rates for the *Salmonella* serovars are shown in **Table 5**. Notably, MDR was widespread serovars Kentucky, Typhimurium, Derby, Agona, and Corvallis isolated from retail meat samples.

**Table 3:** Antibiotic resistance for the most common *Salmonella* serotypes isolated from pork and chicken meats in Guangdong.

Antibiotic	Number (%)							
	Typhimurium (*n* = 84)	Rissen (*n* = 81)	Derby (*n* = 75)	London (*n* = 57)	Agona (*n* = 56)	Corvallis (*n* = 46)	Kentucky (*n* = 38)	Mbandaka (*n* = 34)	Others (n = 144)
AMP	70 (83.3)	63 (77.8)	42 (56.0)	35 (61.4)	9 (16.1)	8 (17.4)	8 (22.2)	0 (0)	60 (41.7)
AMC	2 (2.4)	0 (0)	2 (2.7)	0 (0)	0 (0)	0 (0)	0 (0)	0 (0)	3 (2.1)
CTX	8 (9.5)	1 (1.2)	4 (5.3)	0 (0)	3 (5.4)	4 (8.7)	4 (11.1)	1 (2.9)	15 (10.4)
CAZ	2 (2.4)	1 (1.2)	0 (0)	0 (0)	1 (1.8)	2 (4.3)	0 (0)	1 (2.9)	7 (4.9)
FEP	5 (6.0)	0 (0)	3 (4.0)	0 (0)	0 (0)	1 (2.2)	0 (0)	0 (0)	8 (5.6)
GEN	13 (15.5)	2 (2.5)	27 (36.0)	33 (57.9)	0 (0)	0 (0)	3 (8.3)	1 (2.9)	21 (14.6)
STR	50 (59.5)	16 (19.8)	27 (36.0)	39 (68.4)	9 (16.1)	8 (17.4)	5 (13.9)	25 (73.5)	32 (22.2)
AMK	1 (1.2)	0 (0)	1 (1.3)	0 (0)	1 (1.8)	0 (0)	1 (2.8)	1 (2.9)	3 (2.1)
SXT	23 (27.4)	63 (77.8)	32 (42.7)	40 (70.2)	10 (17.9)	8 (17.4)	26 (72.2)	2 (5.9)	44 (30.6)
S3	75 (89.3)	63 (77.8)	69 (92.0)	44 (77.2)	23 (41.1)	34 (73.9)	35 (97.2)	34 (100.0)	91 (63.2)
NAL	25 (29.8)	6 (7.4)	30 (40.0)	1 (1.8)	11 (19.6)	20 (43.5)	11 (30.6)	1 (2.9)	72 (50.0)
OFX	25 (29.8)	7 (8.6)	32 (42.7)	9 (15.8)	51 (91.1)	40 (87.0)	25 (69.4)	21 (61.8)	65 (45.1)
CIP	11 (13.1)	1 (1.2)	25 (33.3)	0 (0)	1 (1.8)	8 (17.4)	11 (30.6)	1 (2.9)	16 (11.1)
FFC	30 (35.7)	5 (6.2)	34 (45.3)	23 (40.4)	49 (87.5)	25 (54.3)	30 (83.3)	0 (0)	42 (29.2)
CHL	34 (40.5)	7 (8.6)	33 (44.0)	24 (42.1)	50 (89.3)	24 (52.2)	30 (83.3)	0 (0)	43 (29.9)
IPM	1 (1.2)	0 (0)	0 (0)	2 (3.5)	0 (0)	0 (0)	0 (0)	0 (0)	0 (0)
PB	2 (2.4)	1 (1.2)	0 (0)	1 (1.8)	1 (1.8)	1 (2.2)	1 (2.8)	1 (2.9)	4 (2.8)
TET	69 (82.1)	77 (95.1)	61 (81.3)	49 (86.0)	27 (48.2)	37 (80.4)	33 (91.7)	33 (97.1)	77 (53.5)

*Abbreviation: AMP, ampicillin; AMC, amoxicillin-clavulanic acid; CTX, cefotaxime; CAZ, ceftazidime; FEP, cefepime; GEN, gentamicin; STR, streptomycin; AMK, amikacin; SXT, trimethoprim/sulfamethoxazole; S3, sulfisoxazole; NAL, nalidixic acid; OFX, ofloxacin; CIP, ciprofloxacin; FFC, florfenicol; CHL, chloramphenicol; IPM, imipenem; PB, polymyxin B; TET, tetracycline*.

**Table 4:** Multidrug-resistant (MDR) *Salmonella* isolates from retail meats in Guangdong, China.

No. of antibiotics	Number (%) of resistant isolates		
	Chicken (*n* = 302)	Pork (*n* = 313)	Total (*n* = 615)
0	7 (2.3)	20 (6.4)	27 (4.4)
1–3	102 (33.8)	98 (31.3)	200 (32.5)
4–6	130 (43.0)	113 (36.1)	243 (39.5)
7–9	38 (12.6)	50 (16.0)	88 (14.3)
≥10	25 (8.3)	32 (10.2)	57 (9.3)
Resistance ≥ 1	295 (97.7)	293 (93.6)	588 (95.6)
MDR ≥ 3	245 (81.1)	229 (73.2)	474 (77.1)

**Table 5:** Multidrug resistance (MDR) of the most prevalent *Salmonella* serovars isolates from retail meats.

Serovars	Number (%) of antibiotics resistance				
	1–3	4–6	7–9	≥10	Total (resistance ≥ 3)
Typhimurium (84)	20 (23.8)	32 (38.1)	16 (19.0)	12 (14.3)	74 (88.1)
Rissen (81)	26 (32.1)	51 (63.0)	3 (3.7)	1 (1.2)	66 (81.5)
Derby (75)	31 (41.3)	12 (16.0)	11 (14.7)	20 (26.7)	54 (72.0)
London (57)	11 (19.3)	22 (38.6)	19 (33.3)	1 (1.8)	42 (73.7)
Agona (56)	19 (34.0)	31 (55.4)	5 (9.0)	1 (1.8)	53 (94.6)
Corvallis (46)	16 (34.8)	18 (39.1)	9 (19.6)	2 (4.3)	38 (82.6)
Kentucky (38)	5 (13.2)	22 (57.9)	8 (21.1)	3 (7.9)	35 (92.1)
Mbandaka (34)	17 (50.0)	17 (50.0)	0 (0)	0 (0)	32 (94.1)
Braenderup (21)	17 (81.0)	1 (4.8)	1 (4.8)	2 (9.5)	7 (33.3)
Enteritidis (21)	7 (33.3)	13 (61.9)	1 (4.8)	0 (0)	17 (81.0)
Meleagridis (20)	6 (30.0)	6 (30.0)	3 (15.0)	0 (0)	9 (45.0)
Stanley (14)	6 (42.9)	0 (0)	0 (0)	0 (0)	0 (0)
Indiana (12)	0 (0)	0 (0)	0 (0)	12 (100)	12 (100)

### Prevalence of ESBLs-Producing Isolates and ESBLs Genes

Six serotypes were detected among 21 ESBLs-producing *Salmonella* isolates (**Table 6**). The most prevalent serotype was Indiana (7/21, 33.3%) followed by Typhimurium (5/21, 23.8%), Kentucky (3/21, 14.3%), Litchfield (3/21, 14.3%), Derby (2/21, 9.5%), Schwarzengrund (1/21, 4.8%). The resistance profiles are shown in **Table 6**. Eight β-lactamases and ESBLs encoding genes, including *bla_TEM-*1*_*, *bla_TEM-*206*_*, *bla_TEM-*214*_*, *bla_CTX-M-*15*_*, *bla_CTX-M-*55*_*, *bla_CTX-M-*64*_*, *bla_CTX-M-*123*_*, and *bla_OXA-*1*_* were identified in the 21 ESBLs-producing *Salmonella* isolates (**Table 7**). Among them, 42.9% (*n* = 9) were found to harbor *bla_OXA-*1*_*, followed by *bla_CTX-M-*55*_* (5/21, 23.8%), *bla_TEM-*206*_* (4/21, 19.0%), *bla_TEM-*214*_* (4/21, 19.0%), *bla_CTX-M-*123*_* (3/21, 14.3%). Moreover, 100% of ESBLs-producing isolates carried at least one of the β-lactamases and/or ESBLs encoding genes, including *bla_OXA-*1*_* (*n* = 9), *bla_CTX-M-*55*_* (*n* = 5), *bla_CTX-M-*123*_* (*n* = 3), *bla_TEM-*206*_* (*n* = 4), and *bla_TEM-*214*_* (*n* = 4). Seven isolates (33.3% were detected as carrying two β-lactamases and/or ESBLs encoding genes. None of the isolates were positive for *bla_SHV_*, *bla_PSE_*, or *bla_CMY_*.

**Table 6:** *Salmonella* carrying ESBLs encoding genes and corresponding antibiotic resistance profile.

Number of isolates	Sample types	Serotypes	Resistance phenotype	ESBLs genes
7	Pork	Derby	AMP+CTX+FEP+NAL+TET	*bla_CTX-M-*55*_*
	Pork	Derby	AMP+CTX+FEP+NAL+TET	*bla_CTX-M-*55*_*
	Pork	Typhimurium	AMP+CTX+CAZ+FEP+STR+S3+OFX+TET	*bla_TEM-*206*_*
	Pork	Typhimurium	AMP+CTX+GEN+STR+SXT+S3+FFC+CHL+TET	*bla_TEM-*214*_*
	Pork	Typhimurium	AMP+CTX+FEP+STR+SXT+S3+OFX+FFC+CHL+TET	*bla_TEM-*206*,_ bla_CTX-M-*55*_*
	Pork	Typhimurium	AMP+CTX+STR+S3+TET	*bla_TEM-*214*,_ bla_CTX-M-*55*_*
	Pork	Typhimurium	AMP+CTX+FEP+STR+S3+NAL+OFX+CHL+TET	*bla_CTX-M-*55*_*
14	Chicken	Indiana	AMP+CTX+GEN+STR+SXT+S3+NAL+OFX+CIP+FFC+CHL	*bla_TEM-*214*_, bla_OXA-*1*_*
	Chicken	Indiana	AMP+CTX+FEP+GEN+STR+AMK+SXT+S3+NAL+OFX+CIP+FFC+CHL+TET	*bla_CTX-M-*123*_*
	Chicken	Indiana	AMP+CTX+CAZ+FEP+GEN+AMK+SXT+S3+NAL+OFX+CIP+FFC+CHL+TET	*bla_OXA-*1*_*
	Chicken	Indiana	AMP+CTX+FEP+GEN+STR+AMK+SXT+S3+NAL+OFX+CIP+FFC+CHL+TET	*bla_TEM-*1*_, bla_OXA-*1*_*
	Chicken	Kentucky	AMP+CTX+GEN+SXT+S3+NAL+OFX+CIP+FFC+CHL+TET	*bla_TEM-*214*_*
	Chicken	Indiana	AMP+CTX+GEN+STR+SXT+S3+NAL+OFX+CIP+FFC+CHL+TET	*bla_OXA-*1*_*
	Chicken	Litchfield	AMP+CTX+CAZ+FEP+GEN+STR+SXT+S3	*bla_CTX-M-*123*_*
	Chicken	Schwarzengrun	AMP+CTX+GEN+S3+NAL+OFX+CIP+FFC+CHL+TET	*bla_OXA-*1*_*
	Chicken	Indiana	AMP+CTX+CAZ+FEP+GEN+STR+SXT+S3+NAL+OFX+CIP+FFC+CHL+TET	*bla_CTX-M-*64*_, bla_OXA-*1*_*
	Chicken	Indiana	AMP+CTX+CAZ+FEP+GEN+SXT+S3+NAL+OFX+CIP+FFC+CHL+TET	*bla_CTX-M-*15*_, bla_OXA-*1*_*
	Chicken	Litchfield	AMP+CTX+CAZ+FEP+GEN+STR+SXT+OFX+FFC+CHL+TET	*bla_CTX-M-*123*_, bla_TEM-*206*_*
	Chicken	Litchfield	AMP+CTX+GEN+STR+SXT+S3+OFX+FFC+CHL+TET	*bla_TEM-*206*_*
	Chicken	Kentucky	AMP+CTX+S3+OFX+FFC+CHL	*bla_OXA-*1*_*
	Chicken	Kentucky	AMP+CTX+GEN+S3+NAL+OFX+CIP+FFC+CHL	*bla_OXA-*1*_*

*Abbreviation: AMP, ampicillin; AMC, amoxicillin-clavulanic acid; CTX, cefotaxime; CAZ, ceftazidime; FEP, cefepime; GEN, gentamicin; STR, streptomycin; AMK, amikacin; SXT, trimethoprim/sulfamethoxazole; S3, sulfisoxazole; NAL, nalidixic acid; OFX, ofloxacin; CIP, ciprofloxacin; FFC, florfenicol; CHL, chloramphenicol; IPM, imipenem; PB, polymyxin B; TET, tetracycline*.

**Table 7:** Prevalence of *Salmonella* carrying β-lactamase and ESBLs encoding genes (*n* = 21).

Genes		Number of isolates	Percentage of isolates
*bla_TEM_*	*bla_TEM-*1*_*	1	4.8
	*bla_TEM-*206*_*	4	19.0
	*bla_TEM-*214*_*	4	19.0
*bla_CTX_*	*bla_CTX-M-*15*_*	1	4.8
	*bla_CTX-M-*55*_*	5	23.8
	*bla_CTX-M-*64*_*	1	4.8
	*bla_CTX-M-*123*_*	3	14.3
*bla_OXA_*	*bla_OXA-*1*_*	9	42.9

### PFGE

**FIGURE 1:**
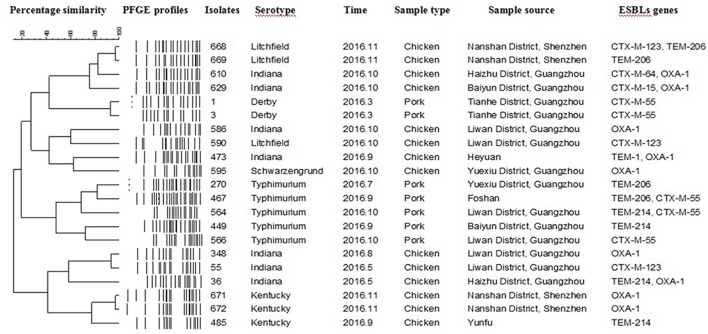
Dendrogram of pulse field gel electrophoresis profiles of 21 extended spectrum β-lactamases (ESBLs)-producing *Salmonella* isolates with the respective ESBL genes from chicken and pork meat.

Twenty-one PFGE patterns were identified among the 21 ESBLs-producing isolates (**Figure 1**). Seven ESBLs-producing Indiana isolates, five Typhimurium isolates, three Kentucky isolates, three Litchfield isolates, two Derby isolates and, one Schwarzengrund isolate were analyzed via PFGE with the enzyme XbaI. The PFGE profile analysis demonstrated that *Salmonella* of the same serotype isolated from different times and locations have markedly different genotypes. Similarly, serovars isolated from the same time and place also showed diverse genotypes.

## Discussion

We found the high levels of *Salmonella* contamination rate in chicken (63.6%) and pork (73.1%) collected from retail markets in Guangdong Province, China. These prevalences were significantly higher than those reported in similar studies in other districts in China, such as Hebei (Yan et al., 2010), Sichuan (Li et al., 2013), Henan (Li et al., 2014), Beijing (Wang et al., 2014), Jiangsu (Bai et al., 2015), and Shandong (Cui et al., 2016). Meanwhile, the *Salmonella* contamination rate was higher than that of other parts of Asia, such as South Korea (Choi et al., 2015), Tokyo (Katoh et al., 2015), and Northern Vietnam (Thai et al., 2012), and higher than that reported in Egypt (Gharieb et al., 2015). This indicated that contamination by *Salmonella* in animal-derived foods in Guangdong Province is more serious, which greatly increases the risk of humans being infected. The results suggested that *Salmonella* in livestock and poultry products should be a priority area for supervision in Guangdong Province. It is also a reminder that animal-derived foods in the tropics should be paid more attention and reflected the need for strengthening of the monitoring of *Salmonella* the animal-origin food supply; for example, improving environmental sanitation in food processing and health awareness among employees should be enhanced, and the disinfection management of processing equipment and tableware containing food should be improved.

Thirty-eight *Salmonella* serovars were found in retail meat samples, among which, serovars Typhimurium, Rissen, Derby (pork), and Agona, Corvallis, and Kentucky (chicken) were the most common serotypes, respectively. The serotypes found in the pork samples were the same as those identified in previous studies in China and abroad (Liang et al., 2015). The distribution of the serotypes in the chicken samples was very different from those in other regions. It was reported that serovars Enteritidis and Typhimurium were two predominant serotypes in chickens from other regions (Li et al., 2017). Interestingly, Agona, Corvallis, and Kentucky were reported less frequently in previous studies (Arnedo-Pena et al., 2016), whereas they were the dominant isolates in the current investigation. Additionally, serovar Agona is one of the top 10 serotypes responsible for nosocomial infections in China (Zhang et al., 2014); serovar Corvallis has been reported in Brazil, Germany, and Turkey (Kocabiyik et al., 2006; Fischer et al., 2013; Yamatogi et al., 2015); and serovar Kentucky was the most common serovar in the United States (van Duijkeren et al., 2002; Galanis et al., 2006). The results suggest that Agona, Corvallis, and Kentucky may cause public health concerns in Guangdong. Importantly, serovar Rumford was detected in animal-derived food for the first time in China. The detection of this strain not only fills the blank of the bacterial species library in China, but also provides a new basis for diagnostic tests and epidemiological investigation of this strain. Notably, Typhimurium is the most frequently isolated serovar from infants in Guangdong (Deng et al., 2012). Therefore, further study is required to determine the source of this serovar (Liang et al., 2015; O’Leary et al., 2015). Our results also showed that an association might exist between *Salmonella*-contaminated food and the sample source. Guangdong is mainly involved in yellow broiler production and its breeding, production mode, and climatic conditions are different from those in other regions, which might explain the different serotype distribution. Thus, China should strengthen yellow chicken monitoring and pay attention to changes in serotypes in different regions.

The susceptibility results showed more than 75% of the *Salmonella* isolates were resistant to tetracycline and sulfisoxazole. These high resistance rates reflect their widespread use in animal feed and are consistent with other reports (Bai et al., 2015). In addition, resistance to florfenicol and chloramphenicol in 47.0% and 48.3% of chicken isolates, in 30.7 and 31.6% of the pork isolates deserves our attention because resistance to these antibiotics may cause human resistance. In this study, all of the *Salmonella* isolates were susceptible to amoxicillin/clavulanic acid and amikacin, possibly because these antibiotics are not used for therapeutic purposes in clinical veterinary medicine; this result was consistent with previous reports (Sinwat et al., 2015). Different serotypes of *Salmonella* showed different resistance rates. In the present study, we observed a high prevalence of MDR *Salmonella* isolates among those isolated from chicken and pork. Serovars Agona, Corvallis, and Kentucky had higher resistance rates to the third-generation cephalosporins (CTX, CAZ) and fluoroquinolones than did the serovars Rissen and Derby. Serovars Agona, Corvallis, and Kentucky also exhibited higher resistance rates to ofloxacin. They also provided more information for further study related to cephalosporin or fluoroquinolones-resistant *Salmonella*. The increasing prevalence of MDR *Salmonella* involving multiple resistances to various antibiotics could cause *Salmonella* to evolve toward becoming a super bacteria (Campioni et al., 2014). The association between antibiotic resistance and specific serotypes should that Kentucky, Typhimurium, Corvallis, and Agona were strongly associated with MDR phenotypes. In addition, resistant strains were found for first line of drug used in human medicine, such as Carbapenems and Polymyxins, which showed that *Salmonella* had a tendency toward drug resistance. Hence, there is a need to strengthen epidemiological investigations of *Salmonella* infections and further studies are required to improve our knowledge concerning the development of MDR.

In the present study, the most commonly detected ESBLs encoding genes were OXAs (42.9%), followed by CTX-Ms (23.8%). This was similar to the results of previous studies in which CTX-M- types were the main family of ESBLs (Nguyen et al., 2016; Qiao et al., 2017). Furthermore, reports indicated that CTX-Ms have been disseminating rapidly among populations of gram-negative bacteria in clinical settings in recent years (Shahid et al., 2009). The 21 ESBLs-producing isolates identified in this study were subjected to PFGE analysis. The resultant dendrograms of isolate patterns showed clusters with a high level of diversity. This indicated that the *Salmonella* prevailing in Guangdong’s retail market during this period not comprised diverse serotypes, but also strains of the same serotype have more complex genotypes and exhibit diverse features. Thus, we should pay attention to this easily overlooked repository of MDR genes, and to the food safety issues they invoke in terms of consumers’ health. Moreover, more research into the characteristics of the dissemination of *Salmonella* and antibiotic resistance genes is warranted.

## Conclusion

Our results indicated the diversity of *Salmonella* serotypes and the high prevalence and antibiotic resistance that existed among *Salmonella* recovered from chicken and pork meat at retail markets. In particular, MDR *Salmonella* serotypes *Agona*, *Corvallis*, and *Kentucky* may indicate the potential risk of resistant *Salmonella* foodborne infections in Guangdong, China. Thus, this study suggests that epidemic trends information about the *Salmonella* serovars in Guangdong province should be further monitored and studied.

## Author Contributions

ML and LZ contributed to the conception of the study. JZ and LZ contributed significantly to analysis and manuscript preparation. LZ performed the data analyses and wrote the manuscript. YF, ZX, and YM helped to perform the analysis with constructive discussions. YW, XQ, and HZ collected the samples and conducted the experiments.

## Conflict of Interest Statement

The authors declare that the research was conducted in the absence of any commercial or financial relationships that could be construed as a potential conflict of interest.

## Footnotes

1 http://www.ncbi.nlm.nih.gov/BLAST/
